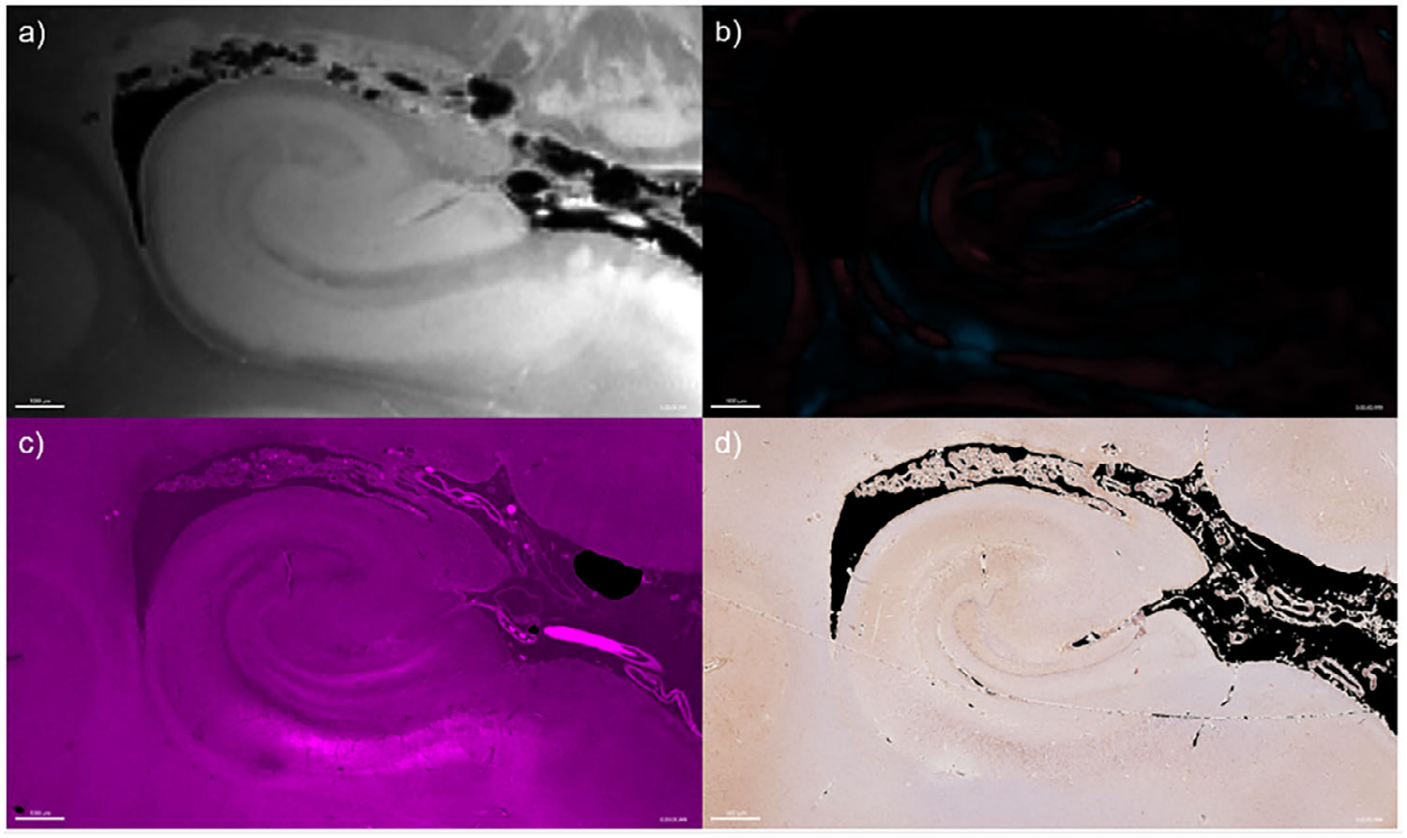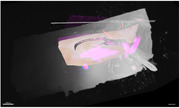# Blood brain barrier leakage in the medial temporal lobe of cases with cerebral amyloid angiopathy

**DOI:** 10.1002/alz70855_098994

**Published:** 2025-12-23

**Authors:** Valentina Perosa, Menab Said

**Affiliations:** ^1^ Massachusetts General Hospital, Harvard Medical School, Boston, MA, USA; ^2^ MassGeneral Institute for Neurodegenerative Disease (MIND), Boston, MA, USA

## Abstract

**Background:**

Quantitative susceptibility mapping(QSM) is a MRI processing technique that relies on gradient‐echo sequences to quantify the magnetic susceptibility of the brain tissue. QSM has been associated to leakage of contrast‐agent in dynamic‐contrast‐enhanced(DCE) MRI and has thus been proposed as a potential marker of blood‐brain‐barrier(BBB)‐leakage^1^. BBB‐leakage is likely involved in the early‐stages of remodeling of blood vessels with cerebral amyloid angiopathy (CAA)^2^, but its prevalence in the MTL of CAA cases remains unexplored. Furthermore, a pathological validation of QSM as a marker of BBB‐leakage is lacking.

**Methods:**

Two autopsy‐cases from a cohort of donors with a clinical diagnosis of CAA were selected. A block of formalin‐fixed brain tissue (∼4x2.5x1cm) from the MTL was scanned at ultra‐high resolution 7 Tesla MRI. The protocol included a T2‐weighted turbo spin echo(TSE) sequence for anatomical reference and a multi‐echo fast‐low‐angle‐shot(FLASH) sequence (voxel‐size 100 μm^3^ isotropic). QSM was calculated from the latter using the open‐source toolbox SEPIA^3^. The tissue‐blocks were embedded in paraffin and the posterior part was additionally imaged using micro‐computer tomography (CT) (voxel‐size 11.86 μm^3^). Subsequently, 6‐μm‐thick serial sections were obtained and each seventh section successively stained for H&E, Perls Prussian blue for iron deposits, and van Kossa for calcium. Immunohistochemistry was performed on adjacent sections for amyloid‐β and fibrin. Whole‐slide sections were imaged with a digital microscope. MRI, micro‐CT and histological sections were aligned to each other using anatomical landmarks of the hippocampus adopting the software Imaris^4^.

**Results:**

Regional (hippocampus and rhinal cortex) measures of QSM and deep‐learning based measures of iron, calcium, and fibrin deposits obtained through Imaris will be compared to each other. Furthermore, three‐dimensional histology will be used to perform a single‐vessel analysis and validate QSM as a measure of BBB‐leakage by correlating perivascular QSM values with corresponding perivascular fibrin‐density. The *ex vivo* study will be complemented by the analysis of *in vivo* MRI data acquired in a cohort of CAA cases (*n* =  14) and control cases (*n* =  7). Regional MTL QSM values will be compared to k‐trans values calculated based on DCE‐MRI.

**Conclusion:**

This study will contribute to validate QSM as a potential, non‐invasive measure of BBB‐leakage.